# Psychometric properties of the geriatric quality of life-dementia in older adults with dementia or mild cognitive impairment living in nursing homes

**DOI:** 10.1186/s12877-019-1307-8

**Published:** 2019-10-22

**Authors:** Eun-Young Park, Seon-Min Park, Jung-Hee Kim

**Affiliations:** 1 Department of Secondary Special Education, College of Education, Jeonju University, PO Box 560-759, 45 Baengma-gil, Wansan-gu, Jeonju, Korea; 2 Department of Clinical Nursing, College of Nursing, The Catholic University of Korea, 222 Banpo-daero, Seocho-gu, Seoul, 06591 Korea

**Keywords:** Dementia, Quality of life, Nursing home, Psychometric properties

## Abstract

**Background:**

Given the theoretical and methodological limitations, there is insufficient knowledge about the psychometric properties and internal structure of quality of life (QOL) measurements for patients with dementia living in nursing homes. The present study aimed to confirm the validity and reliability of the Geriatric Quality of Life-Dementia scale (GQOL-D) to measure the QOL of patients with dementia in nursing homes and analyze their QOL based on the validated GQOL-D factor structure.

**Methods:**

The GQOL-D was used to assess QOL. A convenience sampling method was used to recruit patients with dementia or mild cognitive impairment from six nursing homes in two cities. In order to confirm the validity and factor structure of the scale, both exploratory factor analysis and confirmatory factor analysis were employed. An independent t-test and a one-way analysis of variance were performed to examine the difference in the QOL across general characteristics.

**Results:**

The original factor model was not appropriate to assess the QOL of dementia patients living in nursing homes because the models did not show adequate fit indices. The results support a two-factor structure: environmental and personal factors. Our findings suggest that the internal consistency and construct validity of the proposed two-factor model are adequate, and the GQOL-D is a useful tool for assessing the QOL of dementia patients living in nursing homes.

**Conclusions:**

This factor structure model of environmental and personal aspects is a useful theoretical framework for designing and evaluating interventions for people with dementia and providing integrated person-centered care for people with dementia in nursing homes.

## Background

Owing to the characteristics of degenerative neurological diseases, care and management in long-term care facilities, such as nursing homes, have an important role in the improvement and maintenance of the quality of life (QOL) of individuals with dementia [1, 2]. Assessment of the QOL of individuals with dementia has theoretical limitations, which include lack of conceptual clarity and methodological barriers. QOL is a multidimensional concept, thus making it difficult to define as it is based on individual or disease characteristics in the context of one’s culture and value systems [3, 4].

Measurement of QOL has been recommended as a basic clinical practice [5, 6]. The growing recognition of QOL assessment would enable the provision of holistic and meaningful care to those with cognitive deficits by encouraging healthcare providers to focus more on the individual and less on functional impairments [2, 3]. Many studies have used QOL as an important patient-centered outcome variable for interventions in clinical practice as well as health and social care [7, 8]. The growing emphasis on QOL assessment to understand and improve care provision in healthcare settings [9] should be accompanied with the development of a reliable and valid QOL measure.

Several measurement tools to assess the QOL of people with dementia have been developed. These include the health-related quality of life measure for people with dementia (DEMQOL) [10], Alzheimer Disease Related Quality of Life (ADRQL) [11], Dementia Quality of Life (DQoL) [12], Psychological Well-Being in Cognitively Impaired Persons (PWB-CIP) [13], Quality of Life in Late-Stage Dementia (QUALID) Scale [14], Quality of Life-Alzheimer’s Disease (QOL-AD) [15], Quality of Life Assessment Schedule (QOLAS) [16], QUALIDEM, and Bath Assessment of Subjective Quality of Life in Dementia (BASQID). These tools vary in terms of scale items, types of respondent, method of administration, and the validation process [7, 9]. The common concept of the QOL of individuals with dementia was influenced by Lawton’s model of QOL. According to Lawton [17], QOL of dementia patients involve behavioral competence, patient’s environment, perceived QOL, and psychological well-being. Most of the measures of QOL, such as the QOL-AD, assess the functional and cognitive abilities [18] and focus on the symptoms along with physical and emotional functioning [8]. Other measurements, such as the QUALIDEM, focus on the psychosocial domains of the QOL [19]. Demonstrating the validity of the measurement tool is perhaps the most challenging aspect of scale development. Researchers have addressed this issue in a number of ways to reflect the concept of QOL of patients with dementia [7, 9]. For example, some studies used a disease severity index to examine the validity of a QOL score, indicating that the QOL should decrease as the severity of the dementia increases [20].

Another important aspect of the development and administration of measurement tools is who the respondents are. Due to the subjective nature of QOL, completing the scale items is considered to be challenging for people with moderate-to-severe dementia [21]. Therefore, proxy-rated QOL has been generally used for residents in nursing homes [22–24]. However, there is no consensus on proxy- and self-rated QOL because studies have reported that the reliability of proxy-rating is still questionable for overcoming the barriers to evaluating a subjective concept [3, 8, 25].

Patients with dementia residing in nursing homes had a lower QOL than home-dwelling dementia patients [26]. Although there have been attempts to develop measures—such as survey instruments—to make a quick assessment of QOL, there are still numerous challenges in selecting a valid and reliable self-reported QOL measure for people who have severe impairments that influence their awareness [21].

Moreover, although there are a number of dementia-specific QOL measures, there is insufficient knowledge about the psychometric properties and internal structures of QOL measurements for patients with dementia living in nursing homes [9, 27]. Previous studies conducted for developing measurements reported a difference between the domains of QOL of community dwelling patients and nursing-home patients. One of the reasons for this difference may be that the instrument for measuring the QOL of nursing-home patients focused only on basic activities for minimizing the confounding effects of cognitive impairment [19]. In addition, patients living in long-term care facilities, including nursing homes, tend to be older and at more severe stages of dementia than home-dwelling patients with dementia [28–30].

The Geriatric Quality of Life-Dementia scale (GQOL-D) was developed by including only those items pertaining to QOL that are relevant and important to the Korean older population. The scale’s construct validity and test-retest reliability were confirmed by comparing healthy older adults and dementia patients who visited a clinic, indicating a two-factor structure—namely, psychological and physical environments as well as physical health—which explained 37.7% of the variance [31]. The GQOL-D consists of 15 items and is the most-commonly used measurement to compare the QOL of dementia patients with that of a control group [32–35].

The factor structure of the scale for those living in nursing homes may be different from those living independently in their homes or with their families; therefore, it should be verified for use in nursing homes and research settings. The present study aimed to confirm the validity and reliability of the GQOL-D to measure the QOL of patients with dementia in nursing homes and analyze the QOL of these patients based on the validated GQOL-D factor structure.

## Methods

### Participants

The convenience sampling method was used to recruit patients with dementia or MCI from six nursing homes located in two cities. There are two major recommendations regarding the sample size for the factor analysis. These include an absolute number of at least 200 participants [36] and the subject-to-variable ratio of at least 10 cases for each item in the instrument being used [37]. We analyzed the data of 216 patients with dementia to increase the statistical power.

### Measure

The Geriatric Quality of Life Scale (GQOL) was developed for the general elderly, and further adapted to the Geriatric Quality of Life-Dementia (GQOL-D) for patients with dementia. This tool was applied to the general elderly and dementia patient populations and standardized according to domestic situations. The GQOL-D originally consisted of two factors, namely, psychological and physical environments/physical health [31]. The GQOL-D was used to assess QOL in this study. The GQOL-D has a total of 15 items, with 13 items on physical health, psychological health, independence level, social relations, environment, and religion, one item on overall health, and one item on overall life satisfaction. The clinician asks a question for each item, and the patient indicates their QOL level or satisfaction on each item. Each item is rated on a scale from 1 to 4 (1 = not satisfied; 2 = normal; 3 = satisfied; 4 = very satisfied). The total score ranges from 15 to 60 [31]. In the standardization study, it was confirmed that dementia patients with a K-MMSE (Korean version-Mini Mental Stage Examination) or MMSE-K (Mini Mental Stage Examination Korean version) score of 10 or higher had no difficulty in understanding and responding to the items of the questionnaire [38].

### Procedures

This study used a methodological design to verify the scale’s validity and reliability. The cross-sectional data were obtained from Behavioral and Psychological Symptoms of the Dementia Patient Project, a project for developing an intervention program for improving QOL and reducing the psychological symptoms of dementia. During the 3-year project, patients’ preferred environment for intervention development was investigated, and their quality of life and symptoms of psychological impairment were measured to verify the effect of the intervention program. This study was part of the process for validating the quality of life tools to assess the effectiveness of an intervention program for patients with dementia. Data collection began in June 2018 after obtaining approval from the Institutional Review Board of Catholic University (MC18QNSI0055) to collect data from nursing homes in Korea. The following inclusion criteria were applied to select participants: having a confirmed diagnosis of dementia, a K-MMSE or MMSE-K score of 10 or higher, those aged over 60 years. The K-MMSE or MMSE-K scores were obtained by reviewing each patient’s records at the facilities. It was confirmed that the facilities conduct reexaminations once every 6 months.

Of the 222 participants, 6 participants with incomplete responses were excluded from the study; thus, a total of 216 participants were included in the final analysis. Participants were assured of their anonymity and confidentiality, and informed consent was obtained from all participants. Data were collected by a well-trained research assistant, who administered a questionnaire, which included the GQOL-AD and items on participants’ general characteristics.

### Statistical analysis

In order to confirm the validity and factor structure of the scale, both exploratory factor analysis (EFA) and confirmatory factor analysis (CFA) were employed. First, a CFA was performed for calculating the model fit indices for a two-factor model proposed by a previous study [31]. Then, an EFA was performed for identifying the number of factors and factor loadings. EFA should generally be performed before CFA if there are no strong a priori assumptions about the structure of the factor model that is being tested [39]. Lastly, the CFA was performed again for confirming the construct of the GQOL-D based on the results of the EFA.

The appropriateness of the model was verified by several fit indices in CFA [40]. Incremental fit indices such as comparative fit index (CFI), Tucker-Lewis Index (TLI), and normed fit index (NFI) and absolute fit indices such as chi-square (*χ*^2^) and root mean square error of approximation (RMSEA) was used. RMSEA values < 0.05, 0.06–0.08, 0.08–0.10, and > 0.1 indicate good, reasonable, mediocre, and poor fit, respectively. NFI, CFI, and TLI > 0.90 also indicate a good fit. The Analysis of Moment Structure (AMOS) 20.0 statistical program was used to perform the CFA for obtaining maximum-likelihood estimates of the model parameters and goodness-of-fit indices.

A principle component analysis was used for the factor extraction method and a promax oblique rotation in EFA. Factors were selected based on two criteria: (a) if they had eigenvalues of 1, and (b) scree plot. The scree plot examines the graph to determine the last substantial drop in the magnitude of eigenvalues [41]. The criteria of factor loadings exceeded 0.30 [42, 43]. The Kaiser-Meyer-Olkin Measure (KMO) of sampling adequacy is a statistic that indicates the proportion of variance that may be caused by underlying factors. The KMO value in this study was 0.852, suggesting that a factor analysis is appropriate. In this study, Bartlett’s test of sphericity was significant (*X*^2^ = 1249.177, *df* = 78, *p* = 0.000), also suggesting that a factor analysis is appropriate. Internal consistency reliability of the GQOL-D was assessed by calculating the Cronbach’s α.

An independent t-test and a one-way analysis of variance (ANOVA) were performed to examine the difference in the QOL across general characteristics such as gender, age, marriage status, and education level. The least significant difference was employed for the post hoc analysis. The significance level was set at α = .05.

## Results

### General characteristics of the participants

The general characteristics of the participants are shown in Table 1. A total of 216 patients (52 males and 164 females) with dementia were included in the analysis. The mean age of the participants was 84.10 (SD = 8.00) years, and the mean duration of dementia was 56.45 months (SD = 53.02).

**Table 1:** Characteristics of Patients with Dementia (*n* = 216)

Characteristics	Number	Percent
Gender		
Male	52	24.1
Female	164	75.9
Age		
Below 60	3	1.4
61~70	10	4.6
71~80	46	21.3
81~90	111	51.4
Above 90	44	20.4
Marriage status		
Marriage	71	32.9
Without Spouse	143	66.2
Missing data	2	0.9
Religion		
Yes	145	67.1
No	63	29.2
Missing	8	3.7
Education level		
Below high school	153	70.8
Above high school	52	24.1
Missing data	11	5.1
Presence of child		
No	14	6.5
Yes	202	93.5

The proportion of participants who were married was 32.9%, and the proportion of those without a spouse was 66.2%. Sixty-three participants indicated that they were not religious, whereas 145 participants indicated that they practiced a religion. Regarding participants’ education levels, 52 has graduated high school, whereas 153 had not. Furthermore, 165 participants required aids for mobility, whereas 42 participants did not.

### Confirmatory factor analysis

The two-factor model proposed in a previous study was verified for patients with dementia through the CFA. This model is not appropriate to assess the QOL in patients with dementia because the goodness-of-fit indices were found to be NFI = 0.771, TLI = 0.768, CFI = 0.81, and RMSEA = 1.32.

### Exploratory factor analysis

The results of the EFA are presented in Table 2. The two-factor structure was verified by the EFA, and the two factors explained 54.67% of the variance. Items such as home environment, personal relationship, physical environment, financial resources, positive affect, and recreation/leisure were loaded on the first factor. The remaining items, which included daily activity, mobility, energy, self-esteem, pain/discomfort, memory, and sleep, were loaded on the second factor. As factors are determined based on factor loadings and interpretability, “positive affect” was interpreted as an item of the second factor. The first and second factors were named as environmental and personal factors, respectively.

**Table 2:** Factor structure based on eigenvalue of 1

Items	Environmental	Personal	Communalities
10. Home environment	.845	.305	.420
9. Personal relationship	.811	.393	.625
13. Physical environment	.746	.464	.312
11. Financial resources	.730	.261	.520
4. Positive affect	.662	.583	.364
12. Recreation/leisure	.602	.524	.522
8. Daily activity	.291	.823	.712
7. Mobility	.234	.818	.698
2. Energy	.452	.788	.659
6. Self-esteem	.612	.641	.735
1. Pain/discomfort	.462	.626	.548
5. Memory	.508	.538	.427
3. Sleep	.411	.534	.566
Explained variance (%)	42.549	12.127	54.676

### Confirmatory factor analysis based on the EFA results

The results of verifying the model fit was assumed in CFA, and the specific goodness-of-fit indices are shown in Table 3.

**Table 3:** Fit index of the GQOL-D

*df*	***χ*** ^**2**^	NFI ^a^	TLI ^b^	CFI ^c^	RMSEA ^d^	LO90	HI 90
62	148.452	0.884	0.910	0.928	0.081	0.064	0.097

^a^ Normed Fit Index; ^b^ Tucker Lewis Index; ^c^ Comparative Fit Index; ^d^ Root Mean Square error of approximation

A significant path coefficient was observed for all items (Table 4). Standardized regression weights ranged was from 0.519 (path from memory to personal) to 0.797 (path from home environment to environmental).

**Table 4:** Regression Weights

Path			B^a^	β^b^	SE	C.R.
9. Personal relationship	→	Environmental	1	0.783		
10. Home environment	→	Environmental	0.937	0.797	0.081	11.6^**^
11. Financial resources	→	Environmental	0.688	0.646	0.074	9.271^**^
12. Recreation/leisure	→	Environmental	0.651	0.585	0.078	8.33^**^
13. Physical environment	→	Environmental	0.828	0.724	0.079	10.504^**^
1. Pain/discomfort	→	Personal	1	0.639		
2. Energy	→	Personal	1.079	0.738	0.125	8.634^**^
3. Sleep	→	Personal	0.876	0.544	0.129	6.782^**^
4. Positive affect	→	Personal	1.175	0.708	0.14	8.384^**^
5. Memory	→	Personal	0.846	0.519	0.131	6.466^**^
6. Self-esteem	→	Personal	1.081	0.689	0.132	8.199^**^
7. Mobility	→	Personal	0.865	0.540	0.128	6.74^**^
8. Daily activity	→	Personal	0.878	0.566	0.125	7.011^**^

^**^p < .01. ^a^ unstandardized regression weight, ^b^ standardized regression weight, *SE* standard error, *CR* critical ratio

### The reliability of GQOL-D

The reliability coefficients of the two factors of the GQOL-D are presented in Table 5. The internal consistency of the GQOL-D is excellent, with a Cronbach’s α of 0.909. Cronbach’s α coefficients for environmental factor and personal factors were 0.831 and 0.844, respectively.

**Table 5:** Reliability according to the two factors of the GQOL-D

Factor	Cronbach’s α	95% CI^a^
Environmental	0.831	0.792–0.864
Personal	0.844	0.810–0.873
Total 15 items	0.909	0.890–0.926

^a^Confidence Interval

### QOL level across general characteristics

The mean differences in the QOL scores across participants’ general characteristics are shown in Table 6. The total GQOL-D score was 34.84 (SD = 4.84), which was lower than that of older participants without dementia. The total score of female participants was higher than that of male participants, indicating a higher QOL among female participants (t = − 2.936, *p* < 0.01). For the environmental factor, female participants reported a higher QOL than male participants (t = − 3.944, p < 0.01).

**Table 6:** The level of QOL across general characteristics

Characteristics	Environmental			Personal			Total QOL		
	Mean	SD	*t/F*	Mean	SD	*t/F*	Mean	SD	*t/F*
Gender									
Male	1.88	0.58	−3.944^**^	1.90	0.57	−1.592	28.21	7.51	−2.936^**^
Female	2.29	0.65		2.05	0.59		32.10	8.57	
Age									
Below 70	1.81	0.37	1.702	1.83	0.36	0.527	27.03	5.07	1.159
71~80	2.16	0.73		2.05	0.70		31.43	9.82	
81~90	2.25	0.60		2.03	0.55		31.64	7.94	
Above 90	2.17	0.75		2.01	0.61		30.84	9.06	
Marriage status									
Marriage	2.08	0.72	−1.798	2.02	0.66	0.012	30.48	9.53	−0.881
Without Spouse	2.25	0.62		2.02	0.55		31.57	7.95	
Education level									
Below high school	2.18	0.59	0.253	1.95	0.54	0.990	30.46	7.60	0.817
Above high school	2.21	0.70		2.05	0.61		31.59	8.96	

^**^p < .01, ^*^ *p* < .05

## Discussion

This study aimed to confirm the validity of the GQOL-D to assess the QOL of patients with dementia living in nursing homes and analyze the QOL of these patients based on the validated GQOL-D factor structure. Our findings suggest that the internal consistency and construct validity of the two-factor model are adequate, and the GQOL-D is a useful measure to assess the QOL of dementia patients living in nursing homes. It is critical to measure patients’ QOL using reliable and valid measures, considering cost-effectiveness, and treatment evaluation [3, 16, 44].

The original validation study reported a Cronbach’s α of 0.90 [31], and the results of this study show good indices of internal consistency (0.83–90). It is generally accepted that self-report measures should have an internal consistency reliability coefficient of more than 0.70 and/or 0.80 to be used as a screening tool [45].

This study suggests that the two-factor model is suitable for dementia parents living in nursing homes. The original two-factor model, which consisted of psychological and physical environment and physical health, was not appropriate to assess the QOL of dementia patients living in nursing homes. The proposed factor models were tested by CFA for the entire sample after identifying the factor structure using the EFA. Because there was no strong a priori assumption about the structure of the factor model being tested in the GQOL-D, the EFA was completed before CFA. The results support a two-factor structure: environmental and personal factors.

Compared to the original psychometric testing of the GQOL-D scale, positive effect, self-esteem, and memory, which were included in the first factor (psychological and physical environments of community residing older adults with dementia) [31], were loaded in the second factor (personal factors). Three psychological items showed higher relevance to personal factors in the model. As a result, two distinct factors, environmental factors (personal relationships, home environment, financial resources, and recreation/leisure) and personal factors (pain/discomfort, energy, sleep, positive affect, memory, self-esteem, mobility, and daily activity) were observed.

Generic QOL measures suggest that the concept encompasses the domains of psychological, social. and physical well-being [46]. These domains would not be relevant for the nursing-home patients in this study. The factor structure of QOL among the nursing-home patients comprised both environmental and personal factors. It has been shown that environmental factors such as care provider attitude and communication with staff affect the QOL in nursing-home residents [47]. QOL assessment is defined as a multidimensional evaluation of the person–environment system of individuals in terms of their adaptation to the perceived consequences of dementia [19]. This was in concordance with the QOL measures of the nursing-home patients in this study. The distinct internal structures of QOL measurements for patients with dementia living in nursing homes are due to the clinical differences between community-dwelling patients and nursing-home patients. The community-dwelling participants were identified as individuals aged over 55 and those without cognitive impairment [31]. The average age of individuals living in nursing homes tends to be higher than that of home-dwelling patients with dementia and patients at a more severe stage of dementia [26, 28–30]. Patients with dementia in nursing homes reported greater use of walking aids, less social contact, lower levels of activity, and less exposure to daylight than home-dwelling dementia patients [26]. As age, activities of daily living, and severity of dementia are closely related to QOL in dementia [21], these differences may contribute to the unique factor structures of the scale for dementia patients living in the community and those in nursing homes. In addition, the GQOL-D score of patients with dementia was lower than that of older participants without dementia in this study. Patients with dementia living in residential care should adapt and cope with the physical and psychological environment as the disease progresses [48]; therefore, the environmental factor was revealed to be the first factor.

The QOL scores reported in the present study were lower than those reported in the previous study. Lee et al. [31] conducted a preliminary study to determine the clinical applicability of the GQOL-D. They measured QOL using the GQOL-D in outpatients diagnosed with “dementia of Alzheimer’s disease” or “probable or possible Alzheimer’s disease.” Boredom and loneliness are common among people with dementia in nursing homes due to lack of social contact [49, 50]. Considering the low QOL of patients living in nursing homes, there is a need to develop an intervention program to improve patients’ QOL. Although it has been reported that strategies such as specialized worker approach, training more staff members, and encouraging activity participation in nursing home are important [47], there is insufficient evidence of the intervention program on the improvement of QOL [1]. This factor structure model of environmental and personal aspects proved to be a useful theoretical framework for designing and evaluating interventions for people with dementia and providing integrated person-centered care for patients with dementia living in nursing homes.

Considering that female patients with dementia living in nursing homes were more likely to demonstrate higher ratings of their preference than male patients [49, 51], there may be a gender-related difference in perceiving QOL related to environmental factors. Moreover, as the results regarding the relationships between QOL and socio-demographic characteristics such as gender [31, 52] were inconsistent, role of the gender on QOL of patients living in nursing homes is still unclear. Thus, further studies should examine the gender differences in the self-reported QOL of dementia patients, especially among those living in nursing homes.

This study has some limitations. Possible sampling bias or methodological issues may have affected the interpretation of our data. Participants with MMSE-K scores less than 10 and those who did not complete the questionnaire were excluded from the analysis, and therefore, the study sample may not be fully representative of older adults with dementia living in nursing homes. Thus, evaluating the QOL of patients with dementia remains a challenge, as there might be some construct in this area beyond the conceptual and practical difficulties. Our findings should be compared to those of dementia patients from different clinical settings or at dementia stages. Secondly, the same sample was used in the EFA and CFA to verify the validity of GQOL-D. In this study, we conducted CFA to determine whether the two factors proposed for the general elderly are appropriate for patients with dementia. Because the results of CFA were not appropriate, EFA was performed to identify the factor structure. To confirm the results of the EFA, CFA was conducted again. Therefore, in future studies, CFA should be performed for other samples to confirm the factor structure. Thirdly, we used the MMSE results obtained from data records. Although the facilities have been performing regular reevaluations, it is a limitation of this study that the MMSE score might not reflect recent cognitive function as the time measuring the quality of life. Despite these limitations, the results may enable health professionals and researchers to use this scale and compare treatment efficacy in an experimental study or measure dementia patients’ health outcomes to provide holistic care.

## Data Availability

The dataset used and analyzed during the current study is available from the corresponding author on reasonable request.

## Abbreviations

CFA: Confirmatory factor analysis
EFA: Exploratory factor analysis
GQOL-D: Geriatric quality of life-dementia
QOL: Quality of life
